# Post-cholecystectomy Hepatic Artery Pseudoaneurysm Rupture

**DOI:** 10.7759/cureus.18223

**Published:** 2021-09-23

**Authors:** Mohamed Ahmed, Catherine Fontecha, Marvin Atchison, Rasha Saeed, Kimberly Tustison

**Affiliations:** 1 Surgery, University of California, Riverside, Riverside, USA; 2 Surgery, Riverside Community Hospital, Riverside, USA; 3 Surgery, Memorial Medical Center, Modesto, USA; 4 Surgery, Arrowhead Regional Medical Center, Fontana, USA; 5 Obstetrics and Gynaecology, Temecula Valley Hospital, Temecula, USA

**Keywords:** gastrointestinal bleeding, visceral artery pseudoaneurysm, hepatic artery pseudoaneurysm, laparoscopic cholecystectomy complication, haemobilia

## Abstract

Pseudoaneurysm of the hepatic and/or less frequently the cystic artery is a rare but potentially fatal complication following laparoscopic cholecystectomy. While the procedure is safe with minimal morbidity, complications do occur even in experienced hands. Moreover, patient selection is of utmost importance. These aneurysms usually present with hemobilia a few weeks after surgery; however, free rupture into the peritoneal cavity can occur. Transarterial embolization is the initial management approach when available and feasible. We present a case of a ruptured hepatic pseudoaneurysm three weeks after laparoscopic conversion to open cholecystectomy. The aim is to shed light on this rare but potentially fatal complication.

## Introduction

Approximately 20 million people in the United States have gallstones, and approximately 300,000 cholecystectomies are performed annually. Approximately 10% to 15% of people with gallstones present with symptomatic gallstone disease, which ranges from biliary colic to acute cholecystitis, gallstone pancreatitis, choledocholithiasis, and gallstone ileus [1]. Laparoscopic cholecystectomy is currently the gold standard for the surgical removal of the gallbladder and has distinct advantages of reduced pain, early discharge, and reduced wound complications; however, it is rarely associated with major complications such as vasculo-biliary injury [2].

## Case presentation

**Figure 1:**
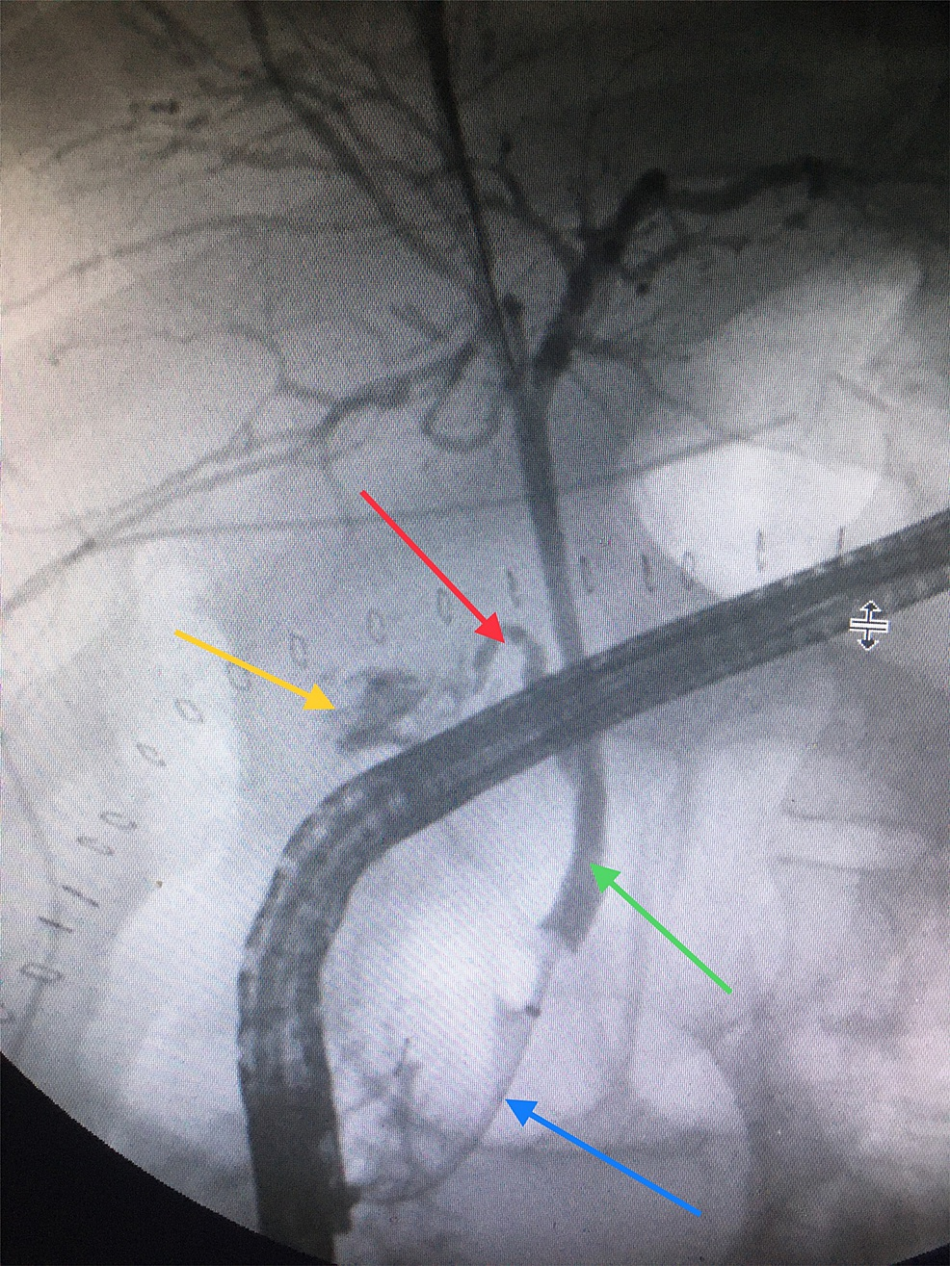
Endoscopic retrograde cholangiopancreatography. Bile leak (yellow arrow), cystic duct (red arrow), common bile duct (green arrow), and stent (blue arrow).

A 53-year-old male with a medical history significant for type 2 diabetes mellitus and hypertension presented to our hospital with a one-week history of epigastric abdominal pain, nausea, and poor oral intake. The evaluation revealed total bilirubin of 6.7 mg/dL (normal: 0-1.1 mg/dL), alkaline phosphatase 242 U/L (normal: 26-137 U/L), aspartate aminotransferase 342 U/L (normal: 0-37 U/L), alanine aminotransferase 598 U/L (normal: 15-65 U/L), glucose 141 mg/dL (normal: 70-100 mg/dL), lipase 10,132 U/L (normal: 73-393 U/L), white blood cell count 12.7 K/μL (normal: 4-11 K/μL), absolute neutrophil 10.2 K/μL (normal: 2-8 K/μL), and C-reactive protein of 79.3 mg/L (normal: 0-9 mg/L). In addition, severe acute respiratory syndrome coronavirus 2 was detected. Computerized tomography (CT) of the abdomen and pelvis was consistent with acute pancreatitis. The assessment was consistent with moderately severe acute pancreatitis [3]. Esophagogastroduodenoscopy with endoscopic ultrasound was performed two days after admission which revealed microlithiasis in the gallbladder, common bile duct stones, and pancreatitis involving the head of the pancreas. Endoscopic retrograde cholangiopancreatography (ERCP) with sphincterotomy was performed with the removal of small stones. Following the procedure, the patient improved. Three days after ERCP, laparoscopic converted to open cholecystectomy with drain placement was performed for a gangrenous gallbladder due to bleeding (1 L blood loss) and failure to progress 45 minutes after the start of the procedure. High-output bile drainage was evident in the early postoperative period. This required a repeat ERCP with stenting for a cystic duct leak 10 days after the surgery (Figure 1), followed by CT-guided drainage of fluid collection.

**Figure 2:**
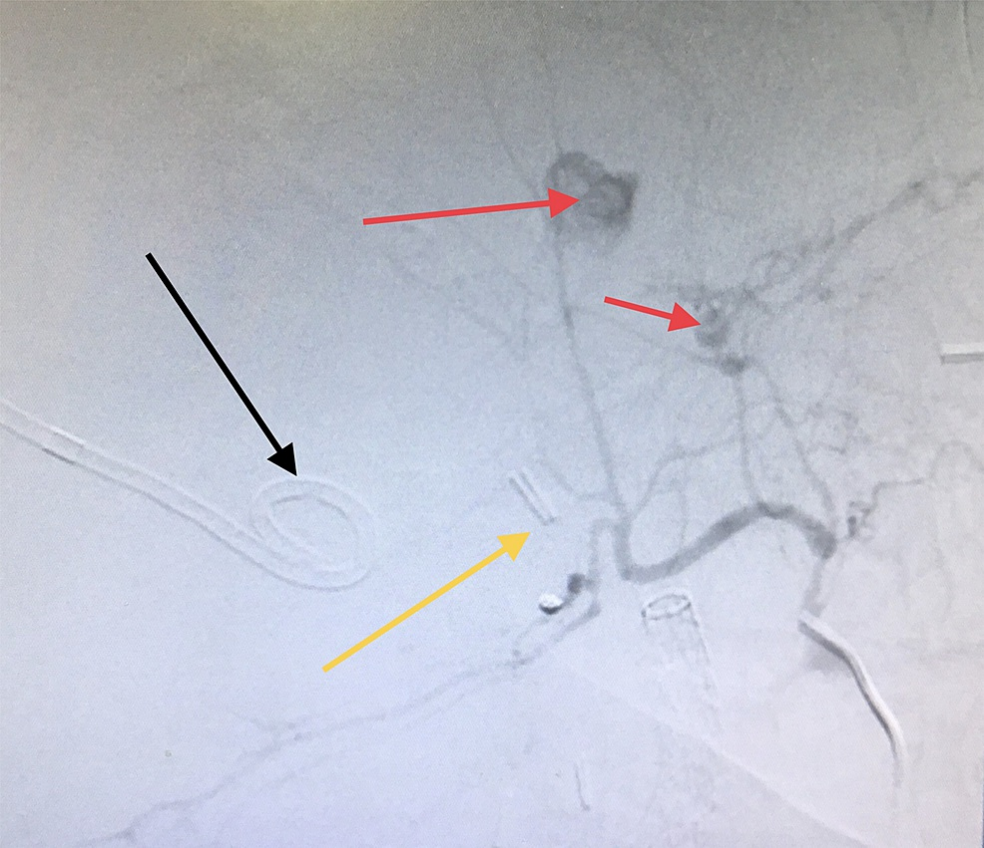
Angiography. Contrast extravasation consistent with active bleeding (red arrows); drain (black arrow); and metal clips used in cholecystectomy (yellow arrow).

The following day, the patient developed melena with a drop in his hemoglobin level. Angiography was performed which revealed multiple foci (most likely multifactorial) of active bleeding from the right hepatic artery (Figure 2).

**Figure 3:**
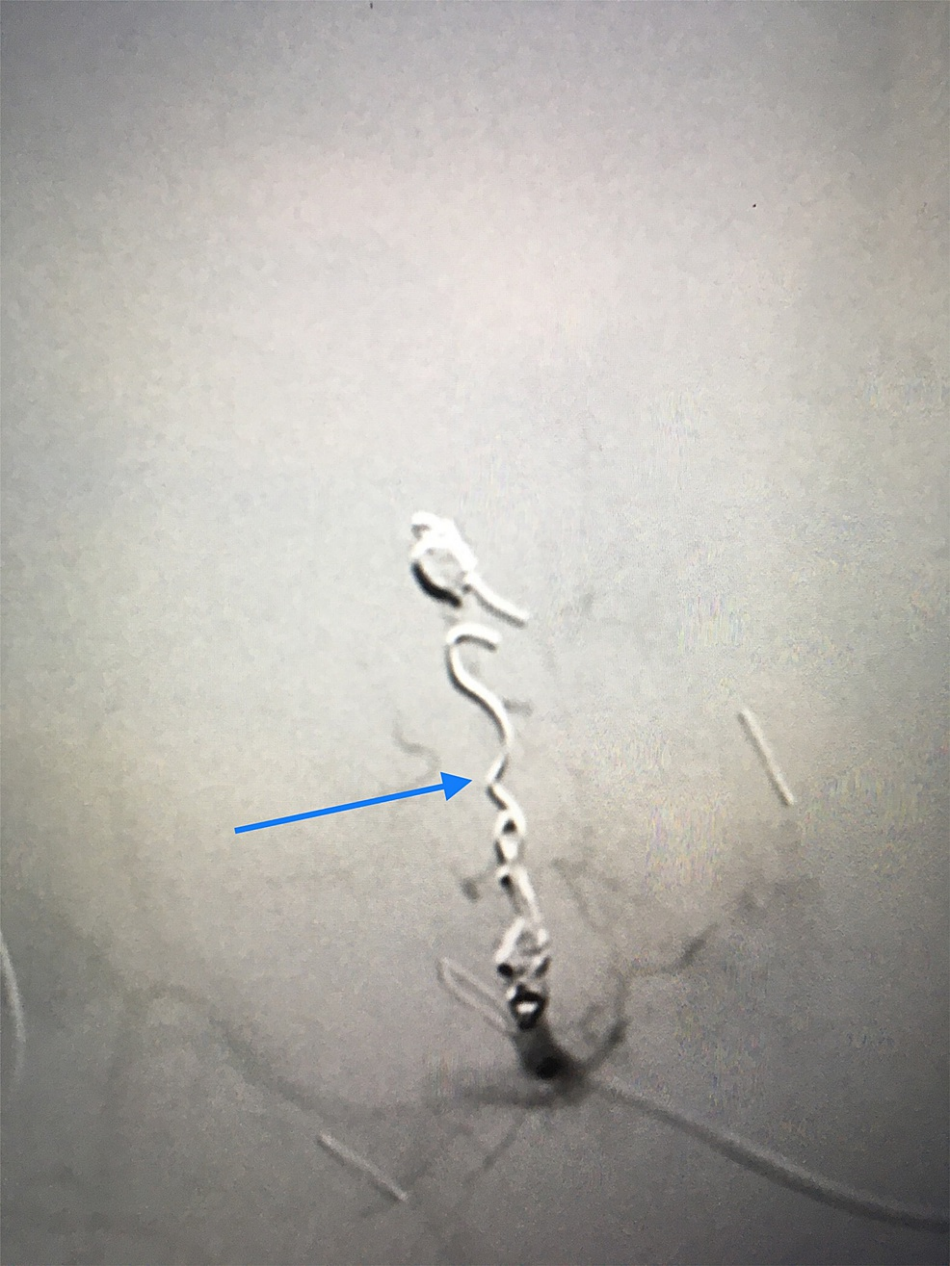
Angiographic embolization of the right hepatic artery. Coils in the right hepatic artery (blue arrow).

The patient was hemodynamically unstable with multiple bleeding sites preventing superselective embolization. Coil embolization of the right hepatic artery was successfully performed with the instant improvement of the hemodynamics (Figure 3).

The patient’s condition rapidly deteriorated with sepsis and renal failure requiring intensive care admission, ventilatory support, and hemodialysis. After six weeks of hospital stay, the patient’s family elected for comfort measures, and support was withdrawn.

## Discussion

Laparoscopic cholecystectomy is a commonly performed safe procedure; however, intraoperative complications can occur, including bleeding from tissues adjacent to the gallbladder (2.83%), bleeding from a cystic artery (0.67%), iatrogenic perforations of the gallbladder (5.27%), injuries to the common bile duct (0.13%), bleeding from the abdominal wall port (1.21%), spilled gallstones (2.02%), and bleeding from the ligaments of the liver (0.54%). Postoperative complications include bleeding from the abdominal cavity at >100 mL/24 hours (3.64%), bile leaks >50-100 mL/24 hours (1.89%), subhepatic collection (0.40%), surgical wound infection (0.94%), incisional hernia (0.40%), hematoma of the abdominal wall (0.67%), retained calculus in the choledochal duct (0.40%), and lost gallstones with abscess formation (0.27%) [4]. Pseudoaneurysms occur due to leakage from an injured artery into the surrounding tissues forming a cavity outside the artery. They can be distinguished from hematoma as they continue to communicate with the arterial lumen resulting in a high-pressure cavity with the risk of rupture [5]. In 1870, Heinrich Quincke described the triad of jaundice, biliary colic, and gastrointestinal bleeding in a patient with a ruptured hepatic artery aneurysm [6]. Pseudoaneurysms of the hepatic and/or cystic artery are a rare complication following laparoscopic cholecystectomy that usually present with gastrointestinal bleeding a few weeks following the procedure. Most patients present with hemobilia (85.1%) with hepatic artery as a source in 88.1%, followed by the cystic artery in 7.9% and a combination of both in 4.0%, with an overall mortality rate of 2.0% [7]. A seven-year retrospective study at the University of California Irvine Medical Center found five cases of hepatic artery aneurysms among 18,015 trauma and surgical admissions (blunt abdominal trauma, liver biopsy, pancreatic pseudocyst, and polyarteritis nodosa), representing an incidence of 0.03%. In addition, there were two cases among 200 orthotopic liver transplants [8]. The use of laser in laparoscopic cholecystectomy was reported as the underlying cause of hepatic artery pseudoaneurysm presenting with hemobilia two weeks after the procedure [9]. Hepatic artery injury is usually associated with bile duct injury [10], and ligation of the hepatic artery is usually safe [11]. In our case, multiple factors could have resulted in the hepatic artery pseudoaneurysm including trauma during a difficult cholecystectomy, infection with abscess formation, pancreatitis, and percutaneous drainage of the abscess. To minimize the morbidity and mortality (as in this case) of cholecystectomy, we encourage the use of the Tokyo guidelines. These guidelines published in 2018 grade acute cholecystitis into three categories. For grade 3, the guidelines recommend urgent biliary drainage followed by delayed laparoscopic cholecystectomy once the patient’s overall condition improves [12].

## Conclusions

Hepatic artery pseudoaneurysm is a rare but potentially fatal complication of a difficult laparoscopic cholecystectomy, usually presenting with gastrointestinal bleeding a few weeks after surgery. Angiography confirms the diagnosis, and transarterial embolization (TAE) is successful in the majority of cases. Surgical ligation of the vessel is required if TAE is unfeasible or fails. TAE is minimally invasive with less morbidity compared to surgical ligation given the hostile surgical field in such cases. Following the Tokyo guidelines flowchart for the management of acute cholecystitis is recommended to minimize the morbidity of laparoscopic cholecystectomy.
